# Laparoscopic resection of a retroperitoneal schwannoma posterior to the renal artery

**DOI:** 10.1016/j.ijscr.2025.111195

**Published:** 2025-03-24

**Authors:** Junhui He, Shuhui Li, Lihua Li, Yulan Gao, Yunji Sun, Lin Cheng

**Affiliations:** a Department of Urology, Heze Municipal Hospital, Heze 274000, China; b Department of Urology, Shandong Provincial Third Hospital, Shandong University, Jinan 250012, China

**Keywords:** Retroperitoneal schwannoma, Retroperitoneal tumor, Clinical pathology, Case report, Diagnosis

## Abstract

**Introduction:**

Retroperitoneal schwannoma is usually asymptomatic, and is often found incidentally during physical examination. It is easy to be misdiagnosed as paraganglioma, retroperitoneal malignant tumor and giant lymphocyte hyperplasia. Accurate preoperative diagnosis is helpful for clinicians to make surgical plans and preoperative preparations. This paper reports a case of retroperitoneal schwannoma. Preoperative imaging suggested a neurogenic tumor behind the left renal artery. The treatment was laparoscopic retroperitoneal tumor resection. The postoperative pathological diagnosis was retroperitoneal schwannoma.

**Case presentation:**

The 52-year-old female patient was admitted to the hospital due to a left retroperitoneal tumor. Preoperative CT and MRI showed neurogenic tumor. After patient authorization, left retroperitoneal tumor resection was performed under laparoscopy. The postoperative pathological diagnosis was retroperitoneal schwannoma. The patient was followed up for 1 year without recurrence or metastasis.

**Clinical discussion:**

The CT features of retroperitoneal schwannoma may be lower than that of the muscle tissue at the same level, with uneven density, cystic lesions, hemorrhage, calcification and other characteristics. Enhanced CT can show progressive enhancement, mild to moderate enhancement in the arterial phase and moderate enhancement in the delayed phase. MRI showed slightly high signal intensity in the center of T2WI lesions, obvious high signal intensity at the edge, and high signal intensity on DWI. The diagnosis of retroperitoneal schwannoma depends on postoperative pathological examination. S-100 protein is one of its specific markers, and most of them are positive. Retroperitoneal schwannoma is mostly benign, but there are also some patients with malignant tumors. The first choice of treatment is complete resection of the tumor, and the tumor margin can also be negative by removing part of the normal adjacent tissue. The survival rate of patients with complete resection can reach 100 %, and the recurrence rate of patients with incomplete resection is about 5–10 %.

**Conclusion:**

Retroperitoneal schwannoma is a relatively rare retroperitoneal tumor. Enhanced CT can show progressive enhancement, mild to moderate enhancement in the arterial phase and moderate enhancement in the delayed phase. MRI showed slightly high signal intensity in the center of T2WI lesions, obvious high signal intensity at the edge, and high signal intensity on DWI. The diagnosis of retroperitoneal schwannoma depends on postoperative pathological examination, and S-100 protein is one of its specific markers. Retroperitoneal schwannoma is mostly benign, but there are also some malignant tumors. The treatment of retroperitoneal schwannoma is the first choice to completely remove the tumor.

## Highlights

• Retroperitoneal schwannoma is usually asymptomatic, and is often found incidentally during physical examination.
• The 52-year-old female patient was admitted to the hospital due to a left retroperitoneal tumor.
• The treatment was laparoscopic retroperitoneal tumor resection, confirmed as retroperitoneal schwannoma.
• The diagnosis of retroperitoneal schwannoma depends on postoperative pathological examination, and S100 protein is one of its specific markers.

## 1 Introduction

Primary retroperitoneal tumors are rare, and most of them are malignant tumors. Schwannoma accounts for about 2 % of the incidence of retroperitoneal tumors. Schwannomas originate from Schwann cells in the peripheral nerve sheath, and are the most common peripheral neurogenic tumors. They are more common in the neck and limbs, and most of them are benign tumors. Retroperitoneal schwannomas are derived from spinal nerve sheath cells, so they tend to occur in areas rich in nerve tissue such as paraspinal, medial kidney and pelvic presacral region, and are closely related to the psoas major muscle [1,2]. Due to the characteristics of the anatomical site, retroperitoneal tumors often grow slowly, and patients are often found by physical examination or other diseases. Patients with symptoms caused by tumor compression of surrounding tissues are rare [3]. The clinical manifestations of primary retroperitoneal tumors are few and lack of specificity, and it is easy to be misdiagnosed as paraganglioma, retroperitoneal malignant tumor and giant lymphocyte hyperplasia. Accurate preoperative diagnosis is helpful for clinicians to make surgical plans and preoperative preparations [4]. We report a case of retroperitoneal schwannoma. Neurogenic tumor was considered by preoperative imaging, and retroperitoneal schwannoma was diagnosed by immunohistochemical staining after operation.

## 2 Case presentation

**Fig. 1:**
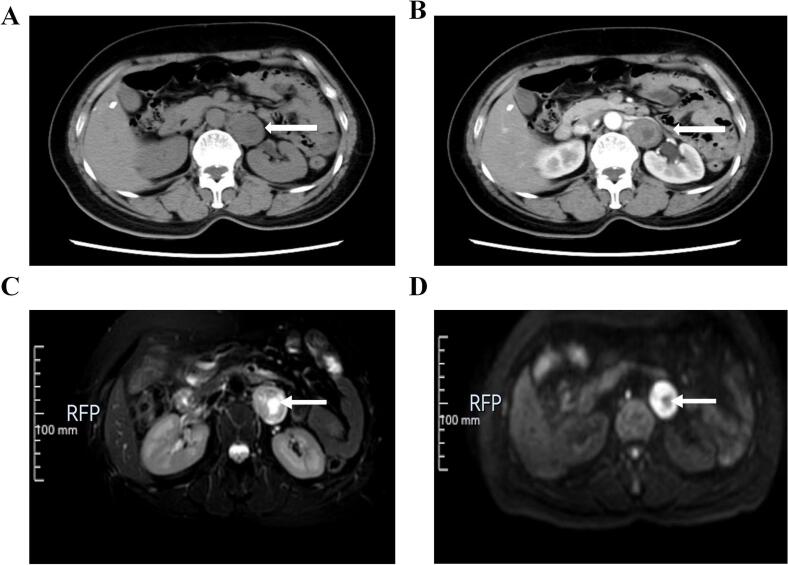
Preoperative imaging images. A. Unenhanced CT of the urinary system revealed a left retroperitoneal mass, 3.2 cm × 2.7 cm in size, with uneven density and a central hypodense lesion; B. Enhanced CT of the urinary system revealed that enhancement was heterogeneous, with no significant enhancement in the central hypodense area of the tumor; C. The results of MRI examination showed slightly high signal intensity in the center of the T2WI lesion; D. DWI showed low signal intensity in the central area of the tumor and high signal intensity in the surrounding area.

**Fig. 2:**
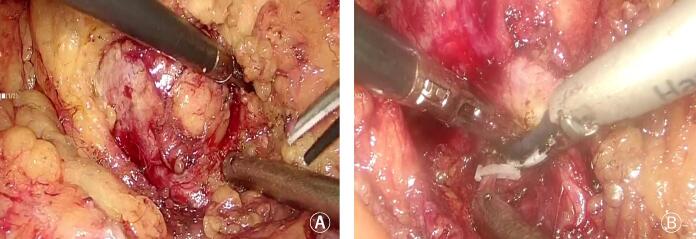
The surgical screenshots of the tumor resection during surgery. A. The tissue around the tumor was separated by ultrasonic scalpel; B. The blood supply vessels were closed by vascular clips.

A 52-year-old female patient with body mass index (BMI) of 20.3 was admitted to the hospital due to “a retroperitoneal mass found on physical examination for 1 day”. The patient was admitted to our hospital after an examination at a community hospital found a retroperitoneal mass 1 day earlier. She had no special medical history. Specialist physical examination revealed no bulging or mass in the bilateral renal region, no tenderness or percussion pain in the bilateral renal region, no bulging or mass. Biochemical and adrenal hormone tests were normal. The possibility of pheochromocytoma could be preliminarily excluded. Unenhanced CT of the urinary system revealed a left retroperitoneal mass, 3.2 cm × 2.7 cm in size, with uneven density and a central hypodense lesion (Fig. 1 A). On the arterial phase, enhancement was heterogeneous, with no significant enhancement in the central hypodense area of the tumor (Fig. 2B). The results of MRI examination showed slightly high signal intensity in the center of the T2WI lesion, significantly high signal intensity at the edge, and high signal intensity in the marginal area of the tumor on DWI (Fig. 2C, D).

## 3 Treatment and outcome

**Fig. 3:**
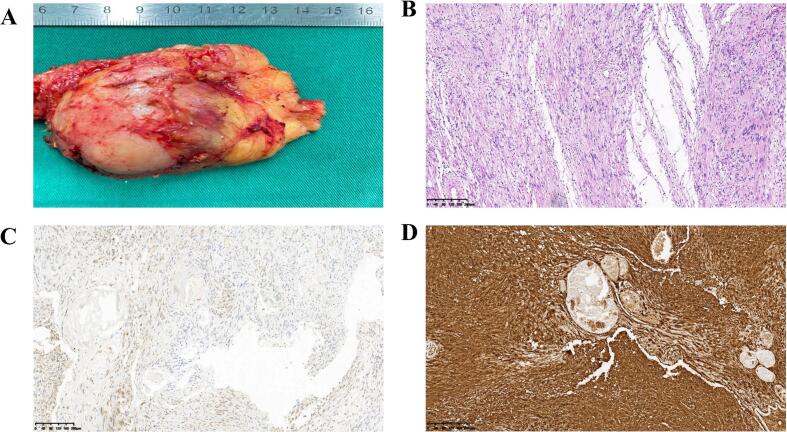
Pictures of pathological specimens and pathological tissue staining pictures. A. Pictures of pathological specimens; B. HE; C. SDHB(+); D. S100(+).

After the patient's authorization and consent for surgical treatment, laparoscopic left retroperitoneal tumor resection was performed. During the operation, a cystic-solid mass, about 4*3 cm in size, was found behind the left renal hilum, which was closely related to the left renal artery and did not invade the renal hilum blood vessels. The tissue around the tumor was separated by ultrasonic scalpel (Fig. 2 A), and the blood supply vessels were closed by vascular clips (Fig. 2 B). The operation time was 1 h and 48 min, and the intraoperative blood loss was about 20 mL. The left retroperitoneal mass was completely removed (Fig. 3 A). Postoperative pathological results: spindle cell tumor, visible cells rich area, the tumor cells are arranged in a palisade pattern (Fig. 3 B). Immunohistochemical: according to S100(+), SDHB (+), CD34 (+), SMA (−), Desmin (−), Dog-1 (−) (Fig. 3 C and D). No recurrence or metastasis during 1 year follow-up. The work has been reported in line with the SCARE criteria [5].

## 4 Discussion

Generally, retroperitoneal schwannoma has no clinical symptoms, and is usually found incidentally during physical examination. When the lesion is large and compresses the surrounding tissue, it can cause nonspecific symptoms such as abdominal pain and low back pain. Therefore, retroperitoneal schwannoma is relatively rare and has no specific clinical manifestations [6]. Retroperitoneal schwannomas are closely related to retroperitoneal nerves. Most of them are located in the retroperitoneal space around the spine, and are relatively common around the kidney. The density of lesions on CT plain scan was lower than that of muscle tissue at the same level, and the density was uneven, which could be accompanied by cystic lesions, hemorrhage, calcification, etc. Enhanced scan showed progressive enhancement, mild to moderate enhancement in arterial phase, moderate enhancement in delayed phase, and the degree of enhancement was diverse. MRI can show isointensity or slightly high signal in the center of the lesion on T2WI, obvious high signal at the edge, and high signal on DWI [7].

Neurilemmoma was microscopically divided into Antoni A and Antoni B areas. Antoni A area: rich in dense spindle cells arranged in a palisade or vortex pattern, not easily cystic, corresponding to solid components in the lesion, generally with moderate to high degree of enhancement. Antoni B area: tumor cells were sparse, arranged in a reticular pattern, with high water content of the stroma, easy to cystic change, bleeding, and generally light or no enhancement. Antoni area A and B may exist alone or in one tumor, so the density of schwannoma is often uneven and prone to cystic degeneration, necrosis, calcification or hemorrhage [8]. The area with slightly high T2WI signal in the central part of the lesion corresponds to Antoni A area. Antoni A area is densely packed with cells and has little water content, so the T2WI signal is isointense or slightly high, while the area with significantly high T2WI signal at the edge corresponds to Antoni B area. Therefore, the T2WI showed significantly high signal intensity. The density and signal intensity of retroperitoneal schwannomas depend on the proportion, arrangement, and distribution of Antoni A and Antoni B areas, and are related to the proportion of fibrous components within the tumor. The typical schwannoma showed progressive delayed enhancement, and the necrotic cystic degeneration area was not significantly enhanced. In different areas of the same tumor, significant enhancement and slight enhancement exist simultaneously [9]. The confirmation of retroperitoneal schwannoma requires pathological examination, and the positive expression of S-100 protein is a unique immunohistochemical feature of schwannoma, confirming that the tumor cells are derived from the neuroectoderm [10].

Retroperitoneal schwannoma is mostly benign, but some patients with malignant tumors are also treated. Complete surgical resection of the tumor is the first choice for treatment, and negative tumor margins can also be achieved by removing part of normal adjacent tissues. The survival rate of patients with complete resection can reach 100 %, and the recurrence rate of patients with incomplete resection is about 5–10 % [11].

## 5 Conclusion

Retroperitoneal schwannoma is a relatively rare retroperitoneal tumor. Generally, it has no clinical symptoms, and is often found incidentally during physical examination. The diagnosis of retroperitoneal schwannoma depends on postoperative pathological examination, and S-100 protein is one of its specific markers. Retroperitoneal schwannoma is mostly benign, but there are also some malignant tumors. The treatment of retroperitoneal schwannoma is the first choice to completely remove the tumor, and negative margins can also be achieved by removing part of normal adjacent tissues.

## Authors' contributions

JHH designed the research study and wrote the manuscript; LC and YJS analyzed and interpreted the data; SHL, LHL and YLG, performed research. All authors reviewed the manuscript. All authors read and approved the final manuscript.

## Consent

Signed informed consent was obtained from the patient for publication of this case report and any accompanying images.

## Ethics approval

The approval was exempt by the Ethical Committee of The Heze Municipal Hospital.

## Sources of funding

Not applicable.

## Patient consent for publication

Signed informed consent was obtained from the patient for publication of this case report and any accompanying images.

## Research registration

Not applicable.

## Declaration of competing interest

The authors declare that they have no competing interests.

## Data Availability

All data generated or analyzed during this study are included in this published article.
